# Short pectus bars with ventral hinge-point stabilization: clinical outcomes in 160 patients

**DOI:** 10.3389/fsurg.2026.1968085

**Published:** 2026-10-29

**Authors:** Anja C. Weinhandl, Werner Schmid, Winfried Rebhandl

**Affiliations:** 1 Department of Pediatric and Adolescent Surgery, Medical University of Vienna, Vienna, Austria; 2 Department of Anaesthesia, Intensive Care Medicine and Pain Medicine, Clinical Division of Pain Medicine, Medical University of Vienna, Vienna, Austria

**Keywords:** entire chest-wall remodeling, hinge-point stabilization, MIRPE, modular stability, multi-bar technique, short-bar technique, ventral stabilization

## Abstract

**Background:**

‘Minimally invasive repair of pectus excavatum’ (MIRPE) was introduced by Donald Nuss in 1998. Numerous modifications have since been proposed to minimize dislocation of the implanted bar(s). Progress was made by utilizing shorter bars, but the underlying biomechanics have remained somewhat unclear. At our center, we routinely use multiple short bars in modular/flexible (as opposed to closed/rigid) constructs with medial unilateral stabilizers, intended to provide freedom both laterally and between bars.

**Methods:**

Having used MIRPE since 2001, we settled for the current approach in 2019. In most cases, we use two or three short bars in parallel, crossed, or “XI” configurations, the latter adding a third bar cranial to the cross. Each bar is inserted through the same retrosternal tunnel and fitted unilaterally with one ventral stabilizer near the thoracic entry point, coinciding with the hinge point of bar rotation. Given no bridging, or additional suturing for fixation, each of the bars in this retrospective series was mechanically independent.

**Results:**

From 07/2019 to 02/2026, this approach was used in 160 consecutive patients. Median age was 16 (IQR: 14–20) years at the time of surgery. Revision due to clinically relevant bar migration was needed in 3 patients (1.88%). Two of these belonged to the minority of 41 adults (≥20 years by WHO criteria), one being a 15-year-old with challenging requirements. Stratified by age groups, the aforementioned rate would translate into 4.88% for adults and 0.84% for children/adolescents. No cases (0%) of bar ‘flipping’ have been observed (exact upper 95% confidence limit: 2.28%).

**Conclusions:**

While fully acknowledging patient-based analysis as the standard, no bar flipping has been seen in 356 mechanically independent, short pectus bars with ventral hinge-point stabilization. Three bars had to be revised due to clinically relevant migration. Comparative studies are needed to determine any advantages that this biomechanical concept may hold over other stabilization strategies.

## 1 Introduction

Of all congenital chest-wall deformities, pectus excavatum has the highest incidence. Its treatment was revolutionized by Donald Nuss in 1998, introducing what we know as ‘minimally invasive repair of pectus excavatum’ (MIRPE) (1). While embraced almost immediately, a long succession of modifications ensued to improve safety and outcomes, and reduce complications, notably in complex cases and in older patients with more rigid chest walls. For example, intraoperative techniques for sternal elevation have minimized the dreaded risk of cardiac or pericardial injury (2–5), and notable recent modifications, too, have remained focused mainly on improving the safety and technical precision of MIRPE (6–9). A historical rundown of techniques that have been developed to prevent bar migration for MIRPE is provided in Supplementary Table S1.

One issue that became apparent early on was a clinically relevant incidence of bar migration that sometimes necessitated reoperation (10–12). Many of these events were caused by rotational instability, and the resultant bar “flipping” became a major concern notably in adolescent and adult patients. The original technique relied on a single long bar with end stabilizers placed bilaterally, and facing sideways, near the mid-axillary line (1, 13). These early concerns about bar migration led to a variety of technical modifications that did succeed in improving stability (2, 4, 13–16). Conceptually, all of them were based on additive fixation of long-bar constructs, including pericostal sutures, multi-point fixation techniques, hinge plates, or other additive means of stabilization.

Progress was made via two, originally separate, strands of development. One was to use bars of reduced length, first reported informally in 2008 (17) and introduced as “short-bar technique” by Pilegaard in 2016 (18, 19). Reduced bar length was not only associated with less dislocation, and flipping in particular, but also facilitated the use of diagonally crossed configurations, effectively doubling support under the deepest point of the deformity for better cosmetic and functional outcomes (18, 19). The other strand was to use multiple bars, both for better stability and to facilitate treatment of complex deformities. Park et al. (20, 21) introduced the concept of morphologically tailored (parallel or crossed) multi-bar configurations to achieve balanced remodeling of the entire chest wall. Other authors, too, reported better outcomes with multiple bars (22, 23). Multiple bars did not, however, *per se* address the problem of rotational instability inherent in long-bar constructs.

The present study expands the scope of our previous report on ’short bars crossed’ in pediatric patients (24). This time around, we shall explain in broader terms why the short-bar technique is such an effective and versatile option for pectus excavatum repair. Light will be shed on the mechanical rationale, configurations, and procedural refinements that have evolved at our department over the years. A case will be made for short bars in various configurations, where each bar is fitted with one unilateral ventral stabilizer, near the entry point to the chest, to achieve hinge-point stabilization.

Judging from the clinical experience at our center, this stabilization technique modifies the fixation method used by traditional MIRPE toward better implant control, force distribution, and overall surgical efficiency. Within the limitations of a case load typical of a single center, a cohort study is presented to document its clinical practicality. A strong focus will be placed on rotational bar stability, but we shall also present other postoperative complications and/or revision requirements, as well as a simple assessment of the cosmetic outcomes and a rundown of the overall surgical approach.

## 2 Materials and methods

### 2.1 Study design, preparations, data collection

In a single-center retrospective study, we analyzed the clinical outcomes of a modified short-bar technique, which is more than the Nuss procedure used with shorter bars, adding specific refinements in bar stabilization and positioning for comprehensive chest-wall remodeling. We have used this approach for all cases of pectus excavatum since 2019, after two decades of routinely using more traditional approaches of MIRPE at our department (Pediatric and Adolescent Surgery, Medical University of Vienna, Austria). Approval of the study protocol was obtained from the institutional review board (EK #2078/2023). Informed consent was not required due to the retrospective design.

For all MIRPE cases during 07/2019–02/2026, data were extracted on demographic variables, preoperative Haller Index (HI), bar configurations, perioperative complications, periods of hospitalization, and postoperative events. Each preoperative assessment included a CT or MRI scan, and photographs had been taken systematically both before and after surgery. Diagnoses of ‘pure’ pectus excavatum accounted for the vast majority of these MIRPE cases, but any ‘mixed’ diagnoses were deliberately excluded anyway to minimize confounders.

### 2.2 Patient positioning, surgical preparations, incisions

After induction of general anesthesia and single-lumen endotracheal intubation, the patient is positioned in a supine position oriented to the right, with the right arm beside the head. Upon careful inspection and palpation of the chest, the deepest point of sternal depression and the surgical entry/exit points are marked using a surgical skin marker. Then a malleable template is fitted to the morphology of the chest wall, based on which the medical-steel bars are shaped. In alignment with the anterior axillary line, 2–3 cm oblique incisions are performed bilaterally (one for single or parallel bars, two for crossed bars) followed by subcutaneous (caudal to pectoral muscle) and submuscular (pectoral muscle or cranial to mamilla) pocket formation to accommodate the stabilizer for each bar.

### 2.3 Thoracoscopy, retrosternal tunneling, vacuum bell

A 5-mm thoracoscopic port is inserted at the mid-axillary line to establish a capnothorax of 10–12 mmHg for improved visualization of the mediastinum, followed by temporary elevation of the sternum using a vacuum bell (3). As shown in Figure 1, a wide mediastinal tunnel is created in the retrosternal space under direct thoracoscopic control, using a vessel-sealing device (LigaSure Maryland; Medtronic, Minneapolis, MN, USA) introduced through a separate right-sided posterior axillary trocar. For controlled bar passage, the dissection is followed by first advancing an introducer from the right-sided entry and hinge point of the prospective bar to its contralateral exit point. Being able to insert multiple bars through a single corridor is but one of several benefits offered by a wide tunnel (see Discussion). In technically demanding cases, an additional trocar may be placed on the left and a blunt dissector introduced to gently hold down the heart during tunnel preparation.

**Figure 1:**
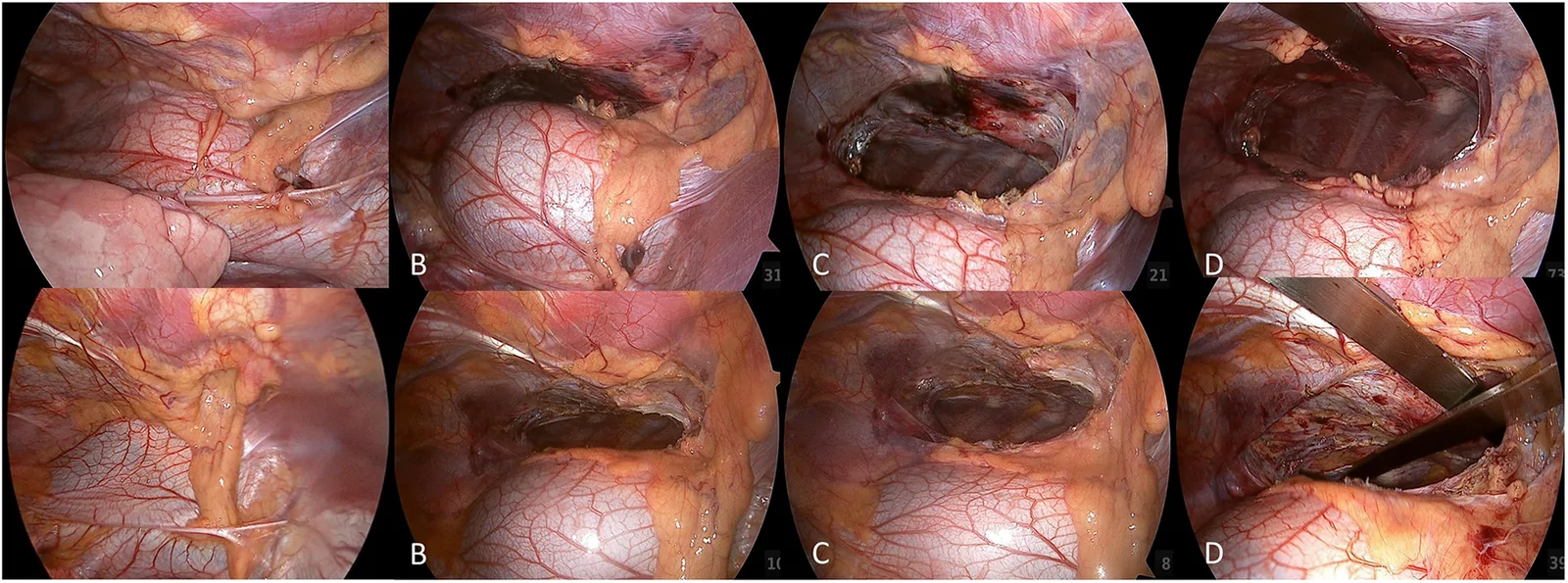
Retrosternal preparation, under thoracoscopic control, of the wide mediastinal tunnel used for the proposed short-bar technique. Two series of photographs are presented, using the same capital-letter inserts to identify intraoperative situations of the same types in different cases. **(A)** Retrosternal space before preparation. **(B)** Situation after tunnel preparation; the vacuum bell was removed for this image to illustrate the tension-releasing effect of the mediastinotomy by itself. **(C)** Sternal elevation with a vacuum bell. **(D)** Situation after bar placement.

### 2.4 Intercostal nerve cryoablation (INC)

Intercostal nerve cryoablation (INC) (25) was introduced in 04/2022 and, ever since, has been routinely applied to the neurovascular bundles under ribs 3–6/ (7) at −70 °C, for 2 min per level, using a cryoprobe (CryoICE; AtriCure, Mason, OH, USA). Percutaneous INC under ultrasound guidance was first introduced as an alternative in 10/2024 and, following a period of selected use cases, has been employed routinely in almost all patients since 06/2025. In these percutaneous scenarios, the probe of a dedicated cryoablation system (Cryo-S-Painless; Metrum Cryoflex, Poland) is applied at least one day prior to surgery.

### 2.5 Bar placement and postoperative follow-up examinations

Each bar is advanced under continuous thoracoscopic guidance, rotated to ventral convexity, and fitted unilaterally with a ventral stabilizer at the thoracic entry point. Surgery is completed by layered wound closure. No chest drains are used. The capnothorax is evacuated and lung recruitment achieved by positive end-expiratory pressure and temporary suction through a catheter before final closure of the drain-free thorax. Postoperative analgesia is provided by INC and 1–2 days of patient-controlled analgesia. Upon discharge, opioid-free pain control is typically maintained by NSAIDs.

Patients were typically seen 1 week after discharge, at around 2 weeks, 1 month, 6 and 12 months, with additional visits as needed until bar removal about 3 years after surgery. A postoperative radiological examination took place to check for relevant pneumothorax or pleural effusion. Another verification x-ray was obtained prior to bar removal. During the follow-up visits in between, the position of each bar was clinically evaluated based on case history, visual inspection, and palpation. An intermediate x-ray for clarification was only obtained if suspicion arose, notably due to recurrence of pain, local protrusion, a change in chest contour, or a conspicuously palpable stabilizer or bar ending. To minimize exposure to ionizing radiation—and considering that the vast majority of patients were children or adolescents—x-rays were not routinely obtained for intermediate visits. While sporadic cases of minor asymptomatic bar dislocation can, therefore, not be categorically ruled out, the ventral orientation of each stabilizer makes it highly unlikely that any clinically relevant bar rotation could have gone unnoticed.

### 2.6 Key considerations and modifications: selecting and configuring the bar(s)

One to three short bars are typically used, four bars may be required in exceptional cases. These implants are manually bent to match the patient's chest-wall anatomy, can be custom-shaped to align with any morphology/deformity, and remain adjustable, in number and orientation, even during the surgical procedure. Length is considered appropriate if a bar extends over at least one rib pair contralateral to the stabilizer. The precise configuration is guided mainly—based on preoperative CT/MRI and intraoperative findings—by sternal-depression size and hemithoracic symmetry. Age can be a useful surrogate for chest wall rigidity. Given low rigidity, or limited deformity, a single bar may do for complete remodeling. In most cases, however, two or three bars will be used in a parallel, crossed, or “XI” configuration (Figure 2).

**Figure 2:**
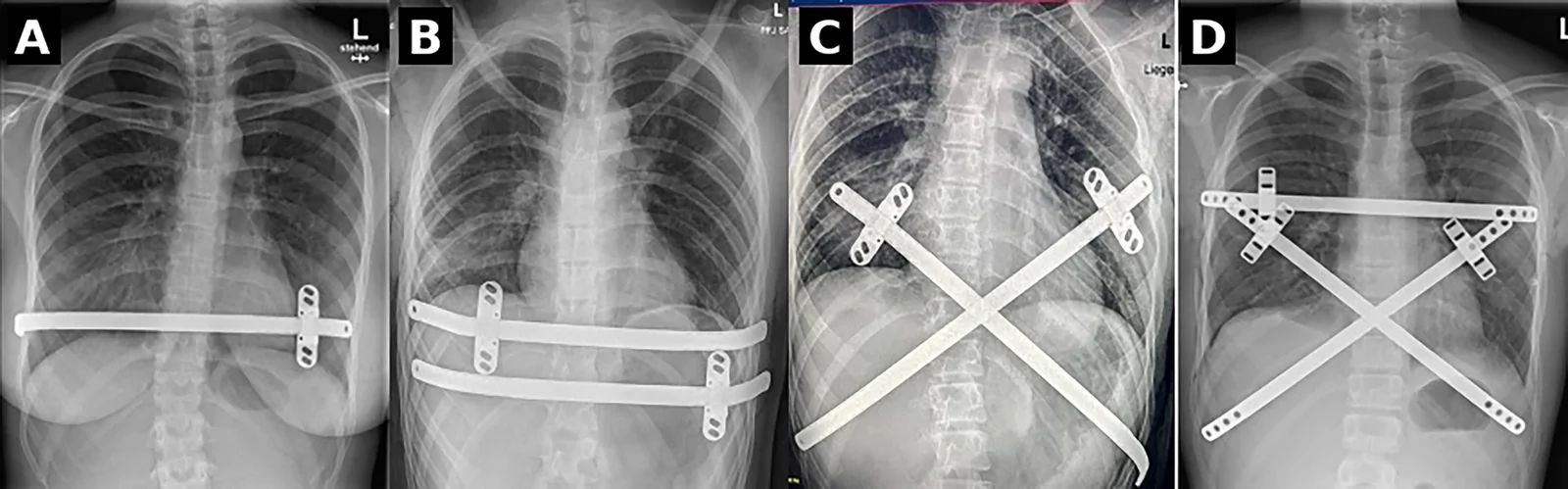
Main configurations of the short bars used in the study. **(A)** Single bar: can effectively correct some wide and symmetrical depressions. **(B)** Two parallel bars (for indication, see A): are inserted horizontally, typically one or two intercostal levels apart; cosmetically advantageous with female anatomy, as the single incision can be hidden inside the inframammary fold. **(C)** Two crossed bars: placed to intersect under the deepest point of the deformity; indicated for short sternum, steep anterior curvature, focal depressions, or focal deformities with rib flaring; vertical and lateral remodeling by force vectors that exert upward pressure centrally and counteracting forces toward the costal arches. **(D)** “XI” configuration: third bar (inserted first, thus elevating the sternum right away to facilitate placement of the crossed bars) for cranial- (manubrial)-oriented deformities or pronounced upper-sternum depressions; enables more complete correction along the cephalocaudal axis.

### 2.7 Key considerations and modifications: stabilizing the bar(s)

Each bar is fitted unilaterally with a single stabilizer that faces ventrally and is located directly at the thoracic entry point, coinciding with the hinge point of the bar (Figures 3A–D). Secure hinge-point fixation is ensured as this stabilizer rests flat on the straight anterior, rather than on the curved lateral, chest-wall area. The intention is to neutralize bar rotation and minimize the risk of migration and flipping (Figure 3E).

**Figure 3:**
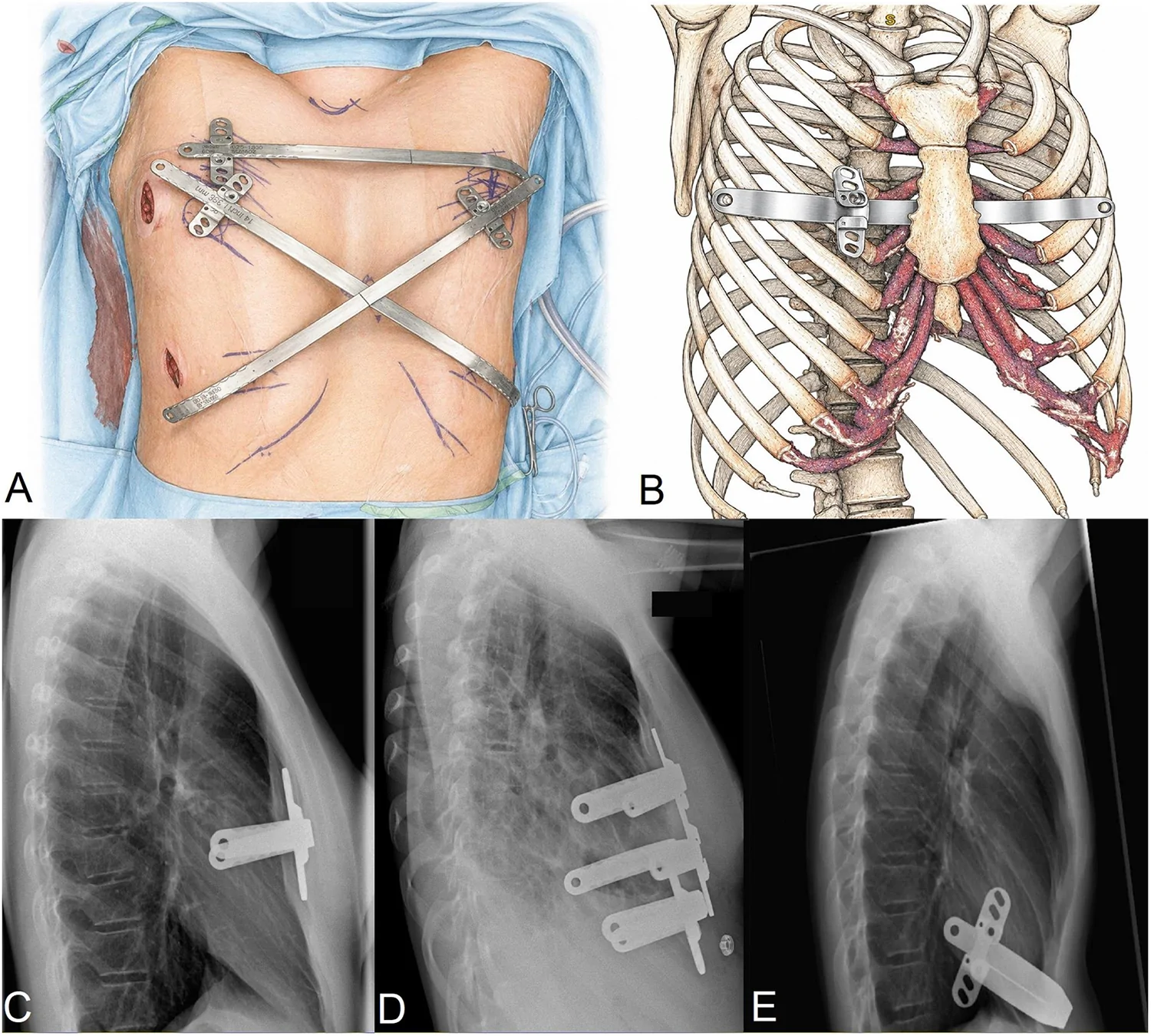
Hinge-point stabilization of bars as proposed in the present study. **(A&B)** One unilateral stabilizer is attached to each bar near the entry point to the chest (AI-enhanced original material). **(C&D)** Thanks to its ventral position, each stabilizer rests flat on the anterior chest wall. **(E)** Our series of 160 consecutive patients included no events of bar ‘flipping’ (*n* = 0).

Short bars are, in and of themselves, biomechanically advantageous over traditional long bars by the very fact that reduced length translates to less leverage, less torque, and less migration. Yet lever forces, mechanical irritation, and seroma formation are reduced further by precise anatomical shaping of the bar and flat-to-flat stabilization against bone at its hinge point. Applied to diagonal cross-bar configurations, this principle of stabilization allows for balanced remodeling of the chest wall in all three dimensions, with elevating forces acting upon the sternum and counteracting forces upon rib flares.

From the earliest patients in this cohort until 10/2024, we used a system (Hipokrat; Hipokrat A.S., Izmir, Turkey) where the stabilizer was firmly secured by custom-bending two kinks along the bar, the first one during anatomical shaping and serving as a stop for the stabilizer, and the second one to secure the stabilizer in its final position. With the current bar system, firm fixation of the stabilizer is ensured by an integrated screw-locking mechanism (Pectus Bar Universal; MedXpert, Eschbach, Germany).

### 2.8 Outcome parameters

The main outcome variable of interest in this study was the incidence of bar flipping due to rotational instability. As additional outcomes, we summarize other revision requirements and/or postoperative complications, durations of surgery, and periods of hospitalization. Bars needing revision because of clinically relevant migration will be distinguished in this report from bars revisited for other reasons. Given requirements of radiation protection (also see the previous subsection explaining the follow-up schedule), a migration event was regarded as clinically relevant only upon radiological confirmation (including, if indicated, by computed tomography) of true malposition in a clinically suspicious, and typically symptomatic, case. A simple (and strictly exploratory) rating system was retrospectively applied to convey a general idea of the cosmetic outcomes, taking into account both the surgeons’ postoperative opinion of complete chest wall remodeling and the patients’ feedback during outpatient follow-up visits. Two patients had been physically lost to follow-up, but their surgical and cosmetic outcomes were adequately and credibly verified by e-mail correspondence.

### 2.9 Statistical analysis

Descriptive statistics are presented. Due to low case numbers in some of the patient subsets of interest, data will mainly be discussed in terms of median values and interquartile ranges. Mean values and standard deviations, while additionally listed, are essentially ‘for information only’. All analysis was performed using spreadsheet software (Excel; v. 2024; Microsoft, Redmond, WA, USA) with a dedicated statistical package (Real Statistics; v. 9.8; www.real-statistics.com). A Kaplan–Meier estimate, covering the time from MIRPE to surgical revision for clinically relevant bar migration, was carried out that involved censoring of patients either at bar removal or, with their bar(s) still *in situ*, at mid-August 2026, truncating the resultant curve when 20% of the cohort remained at risk. On the whole, however, statistical tests were kept to a reasonable minimum to better reflect the descriptive nature of a study intended to present a real-life experience with a specific surgical technique. Also, there was no useful basis for intergroup comparisons regarding the positional stability of bars used in specific configurations or for relevant outcome variables to be compared across studies. Supplementary Table S2 provides details beyond fundamental measures such as *p*-values, whose level of significance was set at 0.05.

## 3 Results

A total of 160 consecutive patients underwent pectus excavatum correction using the short-bar technique with hinge-point stabilization at our center between 07/2019 and 02/2026. Median age at surgery was 16 years (IQR: 14–20), the youngest being 8 and the oldest 55 years. Preoperatively, they exhibited a median Haller Index (HI) score of 5.83 (IQR: 4.55–7.88; Table 1). The 160 procedures involved one bar in 24 (15.0%) and two parallel bars in 63 (39.4%) cases. Crossed bars were used in 73 (45.6%), including a third bar in 56 (35.0%), two bars crossed in 15 (9.38%), or even a fourth bar in 2 (1.25%) cases. Female patients accounted for 15.0% of all cases (*n* = 24), only 8.33% of them (*n* = 2), compared with 52.2% of male patients (*n* = 71), treated by crossed bars (Table 1).

**Table 1:** Patient demographics and Haller index scores at the time of surgery.

Parameter	Category/statistic	One bar		Two bars parallel		Two bars crossed		Three bars (XI)		Four bars (XII)		Total	
		*n* = 24		*n* = 63		*n* = 15		*n* = 56		*n* = 2		*n* = 160	
Patients, *n* (%)	All	24	(15.0%)	63	(39.4%)	15	(9.38%)	56	(35.0%)	2	(1.25%)	160	(100%)
	Male	15	(11.0%)	50	(36.8%)	15	(11.0%)	54	(39.7%)	2	(1.47%)	136	(100%)
	Female	9	(37.5%)	13	(54.2%)	-	-	2	(8.33%)	-	-	24	(100%)
Age, yrs	Median	14.5		16.0		16.0		16.0		46.5		16.0	
	IQR	14.0–18.8		14.0–20.0		14–18.5		14.8–18.0		42.3–50.8		14.0–20.0	
	Range	9–31		8–43		13–39		13–46		38–55		8–55	
	Mean ± SD	16.7 ± 5.20		18.0 ± 6.40		19.9 ± 9.87		17.8 ± 6.50		46.5 ± 12.0		18.3 ± 7.40	
Haller index	Median	5.76		5.81		5.12		6.08		7.10		5.83	
	IQR	4.74–7.31		4.49–7.95		3.98–5.95		4.87–9.30		6.39–7.81		4.55–7.88	
	Range	2.95–11.6		2.90–14.9		3.16–9.50		3.16–39.0		5.68–8.52		2.90–39.0	
	Mean ± SD	6.14 ± 2.21		6.60 ± 2.81		5.31 ± 1.78		8.08 ± 5.94		7.10 ± 2.01		6.95 ± 4.15	

None of the variables shown in this table were normally distributed across the sample (*p* < 0.05; Shapiro–Wilk test). Any conclusions should therefore be drawn based on median values and interquartile ranges (IQRs). Mean values and standard deviations (SDs) should be regarded with caution; here they are provided as strictly auxiliary measures.

### 3.1 Events of clinically relevant bar migration, adolescents vs. adults

A 1.88% patient-based rate of surgical revision due to clinically relevant bar migration was observed. Three patients were affected, each of them accounting for one event (Table 2). These three cases included one three-bar, one two-bar parallel, and one single-bar configuration. Hence a trend for bar dislocation to be associated with specific bar configurations was not apparent. No cases (0%) of bar ‘flipping’ due to rotational instability were observed (exact upper 95% confidence limit: 2.28%).

**Table 2:** Overview of surgery-related complications.^a.^

Timing		Complication	n	%
Intraoperative		Pneumothorax requiring drainage	2	1.25%
Early postoperative		Pleural effusion^b^	7	4.38%
		Local wound complications	9	5.63%
Late postoperative		Surgical revision due to clinically relevant bar dislocation	3	1.88%
		Surgical revision for other positional adjustments^c^	2	1.25%
		Hematothorax	1	0.63%
		Bar infection	2	1.25%
		Metal allergy	2	1.25%
		Pleural effusion^b^	1	0.63%

a Per-event basis (i.e., specific patients may appear once or multiple times).

b Conservative management or drainage.

c To address bar-end curvature (*n* = 1) or unilateral bulging of a bar (*n* = 1).

Stratified by age groups, this 1.88% total rate of surgical revision would translate into 4.88% for adults (2 in 41) and 0.84% for children/adolescents (1 in 119), based on the WHO definition of adolescence covering ages 10 through 19. By this definition, 119 patients in our series were children or adolescents at the time of surgery, while the remaining minority of 41 adults, in turn, included 27 patients in their twenties, 10 in their thirties, 3 in their forties, and 1 in his fifties.

While fully acknowledging patient-based analysis as the standard, it is also true that not a single one of 356 mechanically independent bars has been seen to undergo ‘flipping’ in this series.

### 3.2 Complete versus ongoing treatments, time-to-event analysis

By mid-August 2026, surgical bar removal has been accomplished in 58 of the 160 patients (36.3%). Their bars had been *in situ* for a median of 1,106 (IQR: 1,069–1,185) days. In 102 patients (63.8%), the removal procedure has yet to be performed; these bars have been *in situ* for a median of 630 (IQR: 395–924) days. The revision procedures for the aforementioned three cases of clinically relevant bar migration were performed 11, 35, or 343 days after MIRPE. These findings yield a probability of clinically relevant bar migration (using the Kaplan–Meier method) amounting to 1.25% (95% CI: 0.31–4.91) after 0.5 years and to 1.98% (95% CI: 0.64–6.02) after 1 year up to 3.05 years (1,115 days), which is when 20% (32/160) of the patients continued to be at risk, meaning that they still had their bars *in situ*. The corresponding curve of event-free probability is shown in Figure 4.

**Figure 4:**
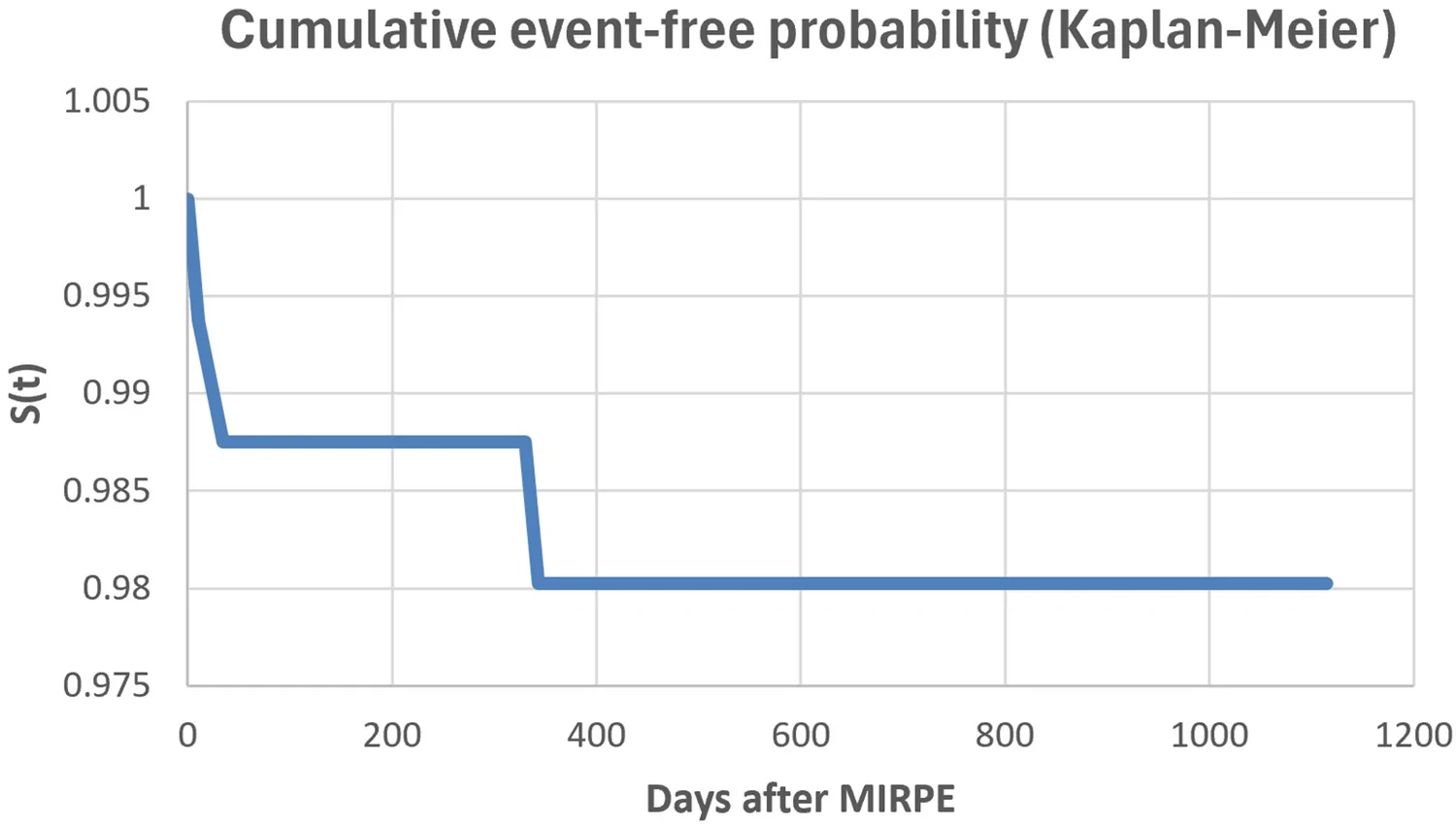
Kaplan–Meier curve illustrating the event-free probability of clinically relevant bar migration, based on days from MIRPE to surgical revision. Numbers of patients at risk after 0.5 years: *n* = 156; after 0.75 years: *n* = 143; after 1.0 years: *n* = 133; after 1.5 years: *n* = 115; after 2.0 years: *n* = 97; after 2.5 years: *n* = 80; and after 3.0 years: *n* = 40. The curve is truncated at 3.05 years after MIRPE based on *n* = 32 patients, representing 20% of all cases (*n* = 160) in this series still at risk.

### 3.3 Other postoperative events

Table 2 summarizes all surgery-related complications on a per-event basis (meaning that specific patients may be included once or multiple times). Most complications were minor, self-limiting, and without long-term sequelae. Events of pleural effusion were managed conservatively or by drainage. Sporadic events of pneumothorax, local wound complications, hematothorax, bar infection, or allergy are not indicative of larger trends.

### 3.4 Durations of surgery, intercostal nerve cryoablation (INC)

Median duration of surgery was 120 (IQR: 90.8–145) min and, as shown in Table 3, increased from one bar (73.5 min) through two parallel bars (109 min), two bars crossed (122 min), three bars (142 min), up to four bars (206 min). Yet the natural agreement between surgical duration and complexity may be exaggerated by INC having been applied intraoperatively during part of the study. While no INC took place in 36 patients (2019–2022; one in 2025), INC was administered intraoperatively (intra-INC) in 88 (2022–2025) and preoperatively (pre-INC) in another 36 (2024–2026).

**Table 3:** Durations of surgery, periods of hospitalization, cosmetic outcomes.

Parameter	Category/statistic	One bar	Two bars parallel	Two bars crossed	Three bars (XI)	Four bars (XII)	Total
		*n* = 24	*n* = 63	*n* = 15	*n* = 56	*n* = 2	*n* = 160
Durations of surgery, min	Median	73.5	109	122	142	206	120
	IQR	50.8–97.3	88.0–134	106–138	118–163	190–223	90.8–145
	Range	37–144	59–193	65–189	66–222	173–239	37–239
	Mean ± SD	78.3 ± 31.4	113 ± 34.2	121 ± 31.2	141 ± 33.5	206 ± 46.7	119 ± 40.2
Periods of hospi-talization, days	Median	3.00	4.00	3.00	4.00	12.5	4.00
	IQR	3.00–4.00	3.00–5.00	3.00–3.50	3.00–5.00	8.8–16.3	3.00–5.00
	Range	2–6	2–9	2–8	3–15	5–20	2–20
	Mean ± SD	3.42 ± 0.88	4.24 ± 1.41	3.60 ± 1.59	4.61 ± 1.90	12.5 ± 10.6	4.29 ± 2.03
Ad-hoc ratings of cosmetic out- comes, *n* (%)	Good/excellent^a^	23 (95.8%)	59 (93.7%)	15 (100%)	51 (91.1%)	2 (100%)	150^a^ (93.8%)
	Mild deficits	1 (4.17%)	2 (3.17%)	-	4 (7.14%)	-	7 (4.38%)
	Moderate deficits	-	2 (3.17%)	-	1 (1.79%)	-	3 (1.88%)
	Severe deficits	-	-	-	-	-	-

a Includes 2 patients lost to follow-up but adequately confirmed (via e-mail correspondence) as uneventful and cosmetically fine.

Of the variables in this table, only the durations of surgery are normally distributed (Shapiro–Wilk test: *p* = 0.464) and, therefore, could be considered primarily based on their mean values and standard deviations (SDs). Normality could not be established for any of the other variables (*p* < 0.05); for them, any conclusions should be drawn based on median values and interquartile ranges (IQRs), while mean values and SDs, being provided as strictly auxiliary measures, should be regarded with caution.

The associated durations of surgery differed significantly both across all three groups (*p* < 0.001) and for pre-INC (79 min; IQR: 67.5–97.0) versus no-INC (127 min; IQR: 89.8–162); for pre-INC versus intra-INC (129 min; IQR: 113–147; 2  ×   *p* < 0.001); but not for no-INC versus intra-INC (*p* = 0.325). This latter finding of no significance, given that the no-INC group overwhelmingly coincides with the earliest cases in this series, presumably reflects a need for procedural/logistic adjustments yet to be completed in this early phase, also considering that this no-INC group was the only INC group to yield a significant result of chronologically decreasing durations of surgery (rho: −0.586; *p* < 0.001).

### 3.5 Lengths of hospital stay, cosmetic outcomes, gender differences

The 160 patients remained hospitalized for a median of 4 (3–5) days. Table 3 lists the distribution of these periods across bar configurations. Differences were again observed in relation to INC, with significant decreases in hospital stay from no-INC (5 days; IQR: 4–6) to intra-INC (4 days; IQR: 3−4) and to pre-INC (3.5 days; IQR: 3-4; *p* < 0.001) but not from intra-INC to pre-INC (*p* = 0.975). In other words, patients whose treatment did involve INC had shorter hospital stays than patients whose treatment did not involve (either intraoperative or percutaneous preoperative) INC.

Based on unvalidated semi-quantitative scores derived *ad hoc* from the non-blinded impressions of two surgeons and patient feedback, the cosmetic outcomes were excellent or good in 150 (93.8%) patients (Table 3). Deficits were mild in 7 (4.38%) and moderate in 3 (1.88%) cases, the latter characterized by overcorrection (*n* = 1), rib flaring (*n* = 1), or residual depression (*n* = 1). A strictly exploratory consistency analysis of two rating runs 2 months apart yielded a good general correlation (rho: 0.869; *p* < 0.001) and intraclass correlation coefficient (ICC: 0.764; 95% CI: 0.688–0.824).

Supplementary Table S3 is provided to account for gender differences. Obviously, caution should be exercised due to a large discrepancy in case numbers (85% versus 15%). However, an association (albeit unadjusted for potential confounders) was observed between longer durations of surgery among the male patients, amounting to a median of 123 (IQR: 92.0–145) versus 105 (IQR: 73.8–131) minutes, and the different bar configurations used with their inherently different levels of surgical complexity (see Tables 1, 3). These durations significantly differed between the sexes (*p* = 0.025) while very similar moderate correlations with bar configurations were observed between the sexes as well (rho: 0.526/525 and 2  ×   *p* < 0.001; see Supplementary Table S2).

## 4 Discussion

The cohort of 160 consecutive patients here presented reflects our real-life clinical experience with short bars in various configurations, each of them fitted unilaterally, near the entry point to the chest, with one ventral stabilizer for hinge-point stabilization. Aside from no events of bar ‘flipping,’ there only were three clinically relevant events of bar dislocation (1.88%).

One of them affected a 15-year-old boy who exhibited the second-highest HI score in this series. He was treated with three bars in an ‘XI’ configuration and experienced a local wound complication (mild swelling in the scar area) in addition to requiring revision, one year or 343 days after surgery, to remove the dislocated upper bar. This boy's cosmetic outcome was rated as excellent. The other two dislocation events occurred in relatively straightforward cases of female patients who, at 23 and 30 years old, did fall into the upper age range of the cohort. Both cases also turned out cosmetically fine and involved early effusion in addition to the revision requirement.

Both the overall 1.88% dislocation rate as such, and its distribution over three completely different bar configurations, following no apparent pattern, support the validity of using short bars with hinge-point stabilization. All three events occurred with the Hipokrat stabilizers we used until 10/2024 (see Methods). The switch to MedXpert was driven by EU regulations, and we have no indications that this could, mechanically or otherwise, have made a difference to the likelihood of bar dislocation.

All three migration events accounting for this overall 1.88% rate were characterized not by long-axis rotation but by horizontal displacement or, more specifically, by contralateral intrathoracic sliding of a bar. All three of these MIRPE procedures had been performed during 2022. Compared with the aforementioned 1-year (343-day) interval between MIRPE and surgical revision in the 15-year-old boy, only 11 or 35 days, respectively, passed until revision in the two (adult) female patients.

As indicated repeatedly in this communication, any short bar used in accordance with our proposed technique should be long enough to span at least one rib pair on its way to the thoracic exit point contralateral to its ventrally oriented stabilizer. The importance of this lesson to preventing such a bar from descending or advancing may not have been sufficiently clear to us in 2022, as apparently the bars in both of the female patients affected fell short of their required length.

### 4.1 Biomechanical considerations

The technique we advocate is relatively easy to implement and has, in our hands, solved major problems of traditional MIRPE related to bar migration and fixation. Few specialized centers seem to have adopted this technique (26), even though the approach continues to be minimally invasive and is fairly well documented (19, 27, 28). In our view, ’short-bar technique’ is not exactly a misnomer but an expression that can result in misconceptions by emphasizing size when the focus should be on hinge-point stabilization. It is true that shorter bars have been associated with less mobility at their endings, better stability overall, less seroma formation, and more lateral freedom for the chest (19, 27, 29).

All these observations, while real, cannot truly explain the excellent performance of short bars, and the same can be said of biomechanical considerations advocating a flat curvature to improve stability (30, 31). Depending on the corrective requirements, flatter bars may actually be unable to transmit adequate force levels, and neither their curvature nor their ’shortness’ are *per se* causative of retrosternal stability, given that bar length, too, remains a functional variable requiring adjustment to age and anticipated growth (29).

The biomechanical principle we are advocating is one of hinge-point stabilization. The unilateral ventral stabilizer needs to be placed such that its wings are well supported by the ribs cranial and caudal to the intercostal entry point. Contralaterally, as has just been noted, it is essential that the bar is prevented from descending or advancing by making sure that it spans at least two ribs on its way to the thoracic exit point. Meeting these requirements should substantially reduce rotational instability and, ideally, obviate the need for additional fixation.

The main source of rotational instability will be eliminated and added fixation no longer needed. Therefore, hinge-point stabilization should be considered a modification to MIRPE in its own right—one that is conceptually distinct, mechanically rational, and offers biomechanical advantages currently not reflected by widespread use in pectus centers (32).

Over the years, a variety of strategies have been developed to prevent bar migration for MIRPE (2, 4, 10, 13, 14, 16, 19, 22, 23). Supplementary Table S1 includes a more detailed historical rundown of these techniques. Many of them tend to increase the invasiveness and hardware burden of MIRPE without addressing the underlying cause of instability, and some of them, like pericostal fixation, should arguably rather be conceived as compensating for the inherent mechanical disadvantages of long bars.

A recent systematic review on bar dislocation after MIRPE found the reported dislocation rates to vary widely (0.9%–33.3%) and identified patient age, chest-wall rigidity, bar length, and placement technique as risk factors (33). Its authors did not, however, focus on minimally invasive stabilization, the short-bar technique, or the underlying biomechanical problems which have led us to use hinge-point stabilization.

### 4.2 Modular stability versus inter-bar bridging

Likewise, no attention was devoted in said review (33) to the biomechanical concept advocated by us, which could be described as ‘modular stability through strategic bar positioning.’ This concept, where each bar will act as a discrete lever addressing a defined segment of the deformity, contrasts with the ‘bridge technique’ introduced by Park et al. in 2015 (15).

Lateral bridging of bars, without direct fixation to bone, is an excellent and well-proven way of effectively preventing bar migration for comprehensive remodeling of complex pectus deformities at a low risk of complications (15, 16, 34–36). Still, a recent clinical review has highlighted that research in this field is characterized by relatively low-level evidence and a lack of formal cost-effectiveness analyses (35). Our present study, while falling into the same category, does suggest that full-coverage reconstruction of the chest wall may also be achievable without bridging and the associated cost considerations.

Large series have shown that multi-bar strategies can work out fine for complex deformities without rigidly interconnected bars (18, 20, 21). Any bridged system will increase what is known as ‘global construct stiffness,’ which can increase primary stability but, at the same time, will reduce the mobility of its individual constituents.

By comparison, multiple bars used in a modular system characterized by hinge-point stabilization will remain mechanically independent. They can be arranged along different craniocaudal levels, each of them selectively bent to address a specific area. Three (in selected cases even four) short bars used in this way can reconstruct even severe deformities while remaining biomechanically discrete.

No evidence is currently available on whether, and to what extent, this concept could make a difference in clinical practice with regard to chest-wall compliance, growth, or physical activity in comparison with bridged systems.

### 4.3 Continuity with the traditional MIRPE procedure

Despite being what we perceive as ‘innovative’ in its own right, modular short bars with hinge-point stabilization remain well within the tradition of MIRPE. Nor is it an entirely new idea to prevent bar instability/dislocation by using shorter bars and redesigned stabilizers placed in a more medial position without fixation wires (37). Likewise, multiple bars have been an option for severe or recurrent deformities all along (38, 39), so the previously introduced ‘bridge technique,’ too, may be considered a modifier, rather than an enabler, of comprehensive chest-wall remodeling (15). Given short bars, in turn, only the parameters of bar length and stabilizer position are modified (18, 19). Even for short bars crossed, the basic procedure of retrosternal passage (rotation to ventral convexity, and stabilization at the hinge point) has not changed from the original approach (1).

Thus the concept we propose could be used in most centers equipped for conventional MIRPE without acquisition expenses for new implant systems or proprietary tools, and without increasing storage/logistic requirements or risking system-specific cost dynamics. Similar considerations apply to its learning curve and surgical safety, as the familiar steps of conventional MIRPE are retained and no bridging hardware or extra suturing is used. That said, we do routinely create a wide mediastinal tunnel in what is basically a complete anterior mediastinotomy from the thymic region to the xiphoid process (see Figure 1). Details on the biomechanical rationale for this step can be found in Weber et al. (40).

### 4.4 Limitations of this research

This study is limited by a retrospective single-center design, no contemporaneous control group, as well as by heterogeneity in patient age, bar configuration, follow-up times, and completion of bar removal. It was not designed to compare fixation systems or to objectively assess chest-wall compliance, growth, physical activity, quality of life, or implementation requirements. Its cosmetic outcomes are reported in an unvalidated fashion based on semi-quantitative scores derived *ad hoc* from the non-blinded impressions of two surgeons and patient feedback; a strictly exploratory consistency analysis is presented, although inter-observer agreement is currently impeded by diverging views on minor residual rib flares.

Since we do not routinely obtain x-rays for follow-up visits, sporadic cases of minor asymptomatic bar dislocation cannot be ruled out with certainty. Yet we consider any optional radiation exposure, notably in children and adolescents, to be unwarranted and the fact of rendering such exposure optional to be actually one more point arguing in favor of using short bars. In contrast to any flipping of long bars with lateral stabilizers facing sideways, we regard the chances of any flipped bar with a ventrally oriented stabilizer escaping our attention as extremely low during clinical examination.

During the study period, changes were implemented in stabilizer design and in the use of intercostal nerve cryoablation. Having discussed potential implications of these changes, we do not see evidence for any real-life effects of these variables on the descriptive validity of our clinical experience or, indeed, on bar migration.

## 5 Conclusions

Our proposed technique of stabilization is about reducing the forces that will act upon, as opposed to reinforce, the pivot of a retrosternal bar implant. Shorter bars getting exposed to lower rotational forces is a proportional function of reduced length but, in addition, also due to a ventral hinge point that causes horizontal loading of bone rather than vertical shearing of intercostal tissue. The rationale of rigidly connecting a stabilizer to each bar in this ventral position is to simplify the hinge-point geometry, decreasing rotational torque (while keeping the bar mechanically independent) and neutralizing the lever ‘trying’ to rotate the bar at its source to minimize the likelihood of bar flipping.

This is how we believe the biomechanical principle of the short-bar technique should be conceptualized, instead of focusing primarily on the length or curvature of these bars. However, it will take comparative studies with biomechanical modeling and clinical outcome analyses to underpin this concept with hard evidence. For the time being, it remains to be seen whether our approach of geometric simplification, or the more traditional approach of reinforcement, is better suited to ensure long-term stability of pectus bars, notably with a view to young patients’ physiological requirements.

## Data Availability

De-identified data supporting the conclusions of this article are available from the corresponding author upon reasonable request, subject to applicable ethical, confidentiality, and data-protection requirements.
