# Supplementary material for: Short pectus bars with ventral hinge-point stabilization: clinical outcomes in 160 patients
Source: Front Surg. 2026 Oct 29;13:1968085. doi: 10.3389/fsurg.2026.1968085 (PMC13755209; doi:10.3389/fsurg.2026.1968085)
Supplement: Supplementary file 1 [file Datasheet1.pdf]

Supplementary Table 1
Modified concepts of bar stabilization for MIRPE.

Year	Author	Short designation	Stabilization concept
1998	Nuss et al. [1]	Original MIRPE	Bilateral stabilizers facing sideways (single long bar)
2001	Hebra et al. [10]	Suture fixation	Pericostal fixation added to prevent dislocation
2004–2008	Park et al. [2, 13]	Mechanism-based fixation	Dislocation analysis → selective fixation techniques
2008	Pilegaard & Licht [17]	Early short-bar application	Reduced bar length
2010	Yoon et al. [22]	Sternal wire fixation	Reinforcement of anterior chest wall
2011	Park et al. [14]	Hinge reinforcement plate	Mechanical stabilization of hinge point
2011	Tedde et al. [35]	Medial stabilizer positioning	Fixation moved toward the midline
2015	Park et al. [13]	Bridge technique	Rigid interconnection of bars
2016	Pilegaard [18, 19, 27]	Short-bar technique	Reduced bar length and more medial stabilization
2016	Jaroszewski et al. [23]	Hammock technique	Suspension-based sternal support
2019–2023	Vinh, Kim et al. [16, 34]	Multi-point/bridge fixation	Increasing reliance on added fixation and rigidity

Reference numbers as used in the main article.

Supplementary Table 2
Overview of statistical tests performed for the various outcome variables.

Probability of clinically relevant bar migration				
Treatment ongoing for	0.5 years	Kaplan-Meier method	1.25%	95% CI: 0.31−4.91
Treatment ongoing for	1 year to 3.05 years*	Kaplan-Meier method	1.98%	95% CI: 0.64−6.02
Durations of surgery depending on sex				
Normal distribution	all patients	Shapiro-Wilk test	p=0.464	
Normal distribution	male patients	Shapiro-Wilk test	p=0.646	
Normal distribution	female patients	Shapiro-Wilk test	p=0.145	
Gender comparison	male versus female	Unequal variances t-test	p=0.025	
Correlations with bar configurations	male patients	Spearman's correlation	p<0.001	rho: +0.526
	female patients	Spearman's correlation	p=0.008	rho: +0.525
Durations of surgery depending on INC				
Normal distribution	INC not performed	Shapiro-Wilk test	p=0.540	
Normal distribution	INC intraoperative	Shapiro-Wilk test	p=0.391	
Normal distribution**	INC preoperative	Shapiro-Wilk test	p=0.010	
Omnibus test**	INC not/intra/pre	Kruskal-Wallis test	p<0.001	
Pairwise intergroup	INC not versus intra	Dunn's test	p=0.325	Holm-Bonferroni adjustment not making a difference
Pairwise intergroup	INC not versus pre	Dunn's test	p<0.001	
Pairwise intergroup	INC intra versus pre	Dunn's test	p<0.001	
Days after 1st application	INC not performed	Spearman's correlation	p<0.001	rho: −0.586
Days after 1st application	INC intraoperative	Spearman's correlation	p=0.333	rho: +0.105
Days after 1st application	INC preoperative	Spearman's correlation	p=0.565	rho: −0.099
Days of hospital stay depending on INC***				
Omnibus test	INC not/intra/pre	Kruskal-Wallis test	p<0.001	
Pairwise intergroup	INC not versus intra	Dunn's test	p<0.001	Holm-Bonferroni adjustment not making a difference
Pairwise intergroup	INC not versus pre	Dunn's test	p<0.001	
Pairwise intergroup	INC intra versus pre	Dunn's test	p=0.975	
Cosmetic outcomes****				
Exploratory consistency analysis only****	First vs. second run	Spearman's correlation	p<0.001	rho: +0.869
	First vs. second run	Intraclass correlation	ICC: 0.764	95% CI: 0.688−0.824

* Patients were censored either at bar removal or, with their bar(s) still in situ, at mid-August 2026, truncating the resultant curve (see Figure 4) when 20% of the cohort remained at risk, which was after 3.05 years (1,115 days).

** Non-parametric due to non-normality of INC-pre; parametric (+post hoc) returned basically the same results.

*** Length of hospital stay was not tested for normality due to its essentially ordinal structure (days).

**** Unvalidated figures based on semi-quantitative scores derived ad hoc from the non-blinded impressions of two surgeons and patient feedback, reported as a strictly exploratory consistency analysis of two rating runs 2 months apart, although inter-observer agreement is currently impeded by disagreement on minor residual rib flares.

INC = intercostal nerve cryoablation.

Supplementary Table 3
Overview of findings grouped by sex/gender.

		Male patients n = 136		Female patients n = 24	
Patients, %		85.0%		15.0%	
Age, yrs	Median	15		17	
	IQR	14–19		14–23.3	
	Range	8–55		11–31	
	Mean ± SD	18.2 ± 7.63		18.8 ± 6.03	
Haller index	Median	5.76		6.41	
	IQR	4.53–7.88		4.94–7.90	
	Range	2.90–39.0		3.27–12.2	
	Mean ± SD	6.96 ± 4.40		6.92 ± 2.51	
Durations of surgery, min	Median	123		105	
	IQR	92.0–145		73.8–131	
	Range	37–239		58–165	
	Mean ± SD	122 ± 40.8		104 ± 33.1	
Periods of hospi-talization, days	Median	4		4	
	IQR	3.00–5.00		3.00–5.00	
	Range	2–20		3–8	
	Mean ± SD	4.31 ± 2.12		4.17 ± 1.40	
Ad-hoc ratings of cosmetic out-comes, n (%)	Good/excellent	127*	(93.4%)	23	(95.8%)
	Mild deficits	6	(4.41%)	1	(4.17%)
	Moderate deficits	3	(2.21%)	-	-
	Severe deficits	-	-	-	-

*Includes 2 patients lost to follow-up but confirmed as cosmetically fine.
